# Acetylated Chitosan Oligosaccharides Act as Antagonists against Glutamate-Induced PC12 Cell Death via Bcl-2/Bax Signal Pathway

**DOI:** 10.3390/md13031267

**Published:** 2015-03-12

**Authors:** Cui Hao, Lixia Gao, Yiran Zhang, Wei Wang, Guangli Yu, Huashi Guan, Lijuan Zhang, Chunxia Li

**Affiliations:** Shandong Provincial Key Laboratory of Glycoscience and Glycoengineering, and Key Laboratory of Marine Drugs of Ministry of Education, School of Medicine and Pharmacy, Ocean University of China, Qingdao 266003, China; E-Mails: haocui2010@hotmail.com (C.H.); xialigao1214@163.com (L.G.); zhangyiran_ouc@126.com (Y.Z.); wwwakin@ouc.edu.cn (W.W.); glyu@ouc.edu.cn (G.Y.); hsguan@ouc.edu.cn (H.G.)

**Keywords:** PC12, peracetylated chitosan oligosaccharide, neuroprotective, reactive oxygen species, apoptosis, mitochondrial membrane potential

## Abstract

Chitosan oligosaccharides (COSs), depolymerized products of chitosan composed of β-(1→4) d-glucosamine units, have broad range of biological activities such as antitumour, antifungal, and antioxidant activities. In this study, peracetylated chitosan oligosaccharides (PACOs) and *N*-acetylated chitosan oligosaccharides (NACOs) were prepared from the COSs by chemcal modification. The structures of these monomers were identified using NMR and ESI-MS spectra. Their antagonist effects against glutamate-induced PC12 cell death were investigated. The results showed that pretreatment of PC12 cells with the PACOs markedly inhibited glutamate-induced cell death in a concentration-dependent manner. The PACOs were better glutamate antagonists compared to the COSs and the NACOs, suggesting the peracetylation is essential for the neuroprotective effects of chitosan oligosaccharides. In addition, the PACOs pretreatment significantly reduced lactate dehydrogenase release and reactive oxygen species production. It also attenuated the loss of mitochondrial membrane potential. Further studies indicated that the PACOs inhibited glutamate-induced cell death by preventing apoptosis through depressing the elevation of Bax/Bcl-2 ratio and caspase-3 activation. These results suggest that PACOs might be promising antagonists against glutamate-induced neural cell death.

## 1. Introduction

Neurodegenerative disease is a general term used to describe a wide range of conditions, such as Alzheimer’s disease (AD) and Parkinson’s disease, and its occurrence increases with advanced age [1]. A large number of evidence indicates that oxidative stress induced by reactive oxygen species (ROS) plays an important role in neurodegenerative disease. ROS are normal byproducts of aerobic respiration and their level is strictly controlled by various cellular antioxidant compounds and enzymes, while their overproduction leads to cell death [2]. Accordingly, tackling free radicals offers a promising therapeutic target in neurodegenerative disease.

Glutamate is one of the major endogenous excitatory neurotransmitters, which plays an important physiological role in the central nervous system [3]. However, in a variety of pathologic conditions, accumulated high concentrations of glutamate can lead to neuronal injury and cell death through two different mechanisms. One of the mechanisms is that glutamate-induced toxicity is mediated by competitive inhibition of cystine uptake, which leads to oxidative stress [4,5,6]. Another mechanism is that the excitotoxicity of glutamate is mediated by several types of excitatory amino acid receptors resulting in a massive influx of extracellular Ca^2+^ [7,8]. It is predictable based on both mechanisms that proper antagonists would be able to prevent glutamate-induced neural injury and cell death. It has been reported that high concentration of glutamate induces PC12 cell death [9,10,11,12] and different types of classical antidepressants could prevent excitotoxicity of activated glutamate receptors in PC12 cells [13]. Therefore, PC12 cells represent an ideal cell-based system to search for antagonists against glutamate-induced cell death and to study underlying molecular mechanisms.

Chitosan oligosaccharides (COSs) are a degradation product of chitosan, which is derived from deacetylation of chitin, the main component of the exoskeleton of crustaceans. A large number of studies have shown that the COSs have various biological activities, including antioxidant, antimicrobial, and antitumor activities [14]. Recently, it has been reported that the COSs possess good neuroprotective properties such as β-amyloid and acetylcholinesterase inhibitory activities, anti-neuroinflammation, and anti-apoptosis effects [15,16,17,18], which suggest the COSs and their derivatives might merit further investigation as novel antagonists against glutamate-induced cell death. 

To accomplish this goal, peracetylated chitosan oligosaccharides (PACOs) and N-acetylated chitosan oligosaccharides (NACOs) were prepared and their effects as antagonists against glutamate-induced cell death and underlying molecular mechanisms were investigated in PC12 cells. The results indicated that the PACOs pretreatment markedly inhibited PC12 cell death induced by glutamate exposure in a concentration-dependent manner. Moreover, pretreatment with the PACOs also significantly reduced lactate dehydrogenase (LDH) release and ROS production. It also attenuated the loss of mitochondrial membrane potential (MMP). Further studies indicated that the PACOs inhibited glutamate-induced cell death by preventing apoptosis through depressing the elevation of Bax/Bcl-2 ratio and caspase-3 activation. These results suggest that PACOs might be promising antagonists against glutamate-induced neural cell death.

## 2. Results and Discussion

### 2.1. Characterization of Chitosan Oligosaccharides and Its Acetylated Derivatives

**Figure 1:**
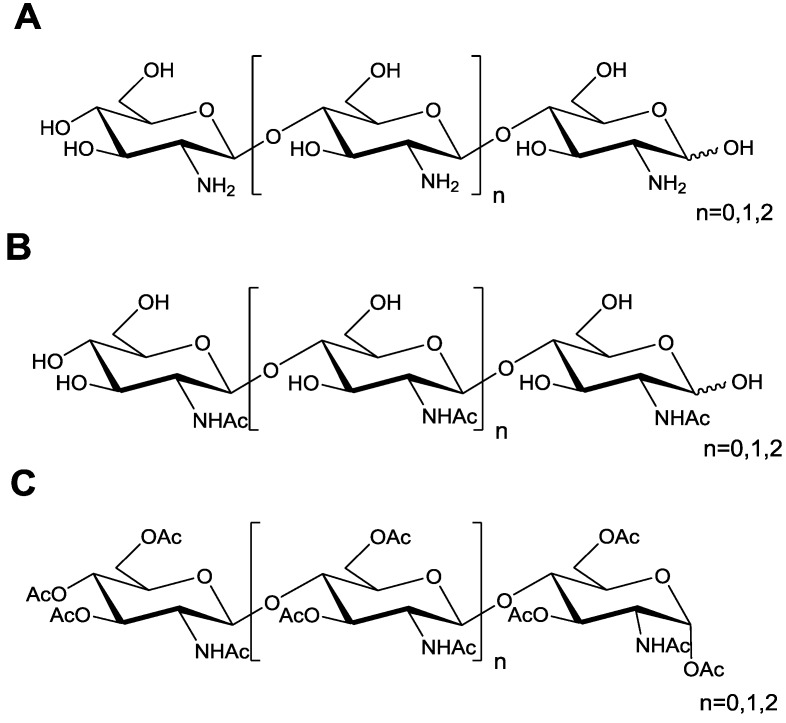
Schematic diagram of the repeating saccharide units in chitosan oligosaccharide and its acetylated derivatives. (A–C) Chemical structure of chitosan oligosaccharides (COS) (**A**), *N*-acetylated chitosan oligosaccharides (NACO) (**B**), and peracetylated chitosan oligosaccharides (PACO) (**C**). The degree of polymerization (DP) of COS, NACO, and PACO is 2~4.

Chitosan oligosaccharide COS was prepared by enzymatic hydrolysis of chitosan as described previously [19]. The COSs had the degree of polymerization (DP) of 2, 3, and 4, respectively. They were named COS-2, COS-3 and COS-4 (Figure 1). The acetylated derivatives of COS (*N*-acetylated chitooligosaccharide (NACO) and peracetylated chitooligosaccharide (PACO)) were prepared using the methods described previously [20]. The structures of Q-2, Q-3, Q-4 (PACOs) and N-2, N-3, and N-4 (NACOs) were also shown in Figure 1. The oligosaccharide structures were confirmed by ^1^H NMR, ^13^C NMR, and MS analyses.

### 2.2. Glutamate-Induced PC12 Cell Death

**Figure 2:**
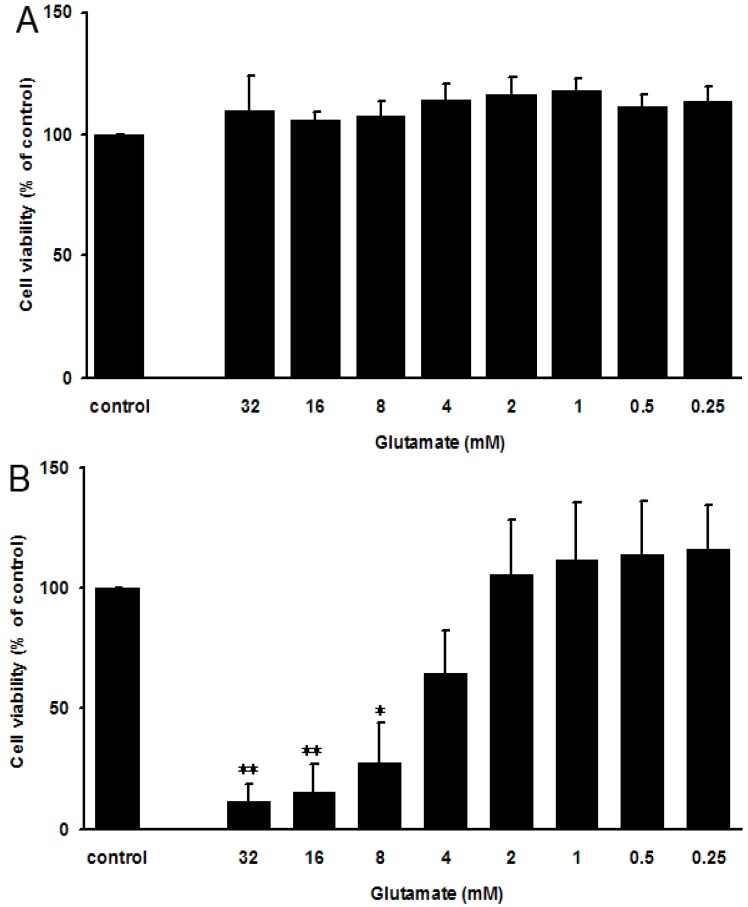
Effect of glutamate on undifferentiated and fully-differentiated PC12 cells. (A–B) The undifferentiated (**A**) and fully-differentiated (**B**) PC12 cells were plated on the cell culture plates at a density of 1 × 10^5^ cells/mL, and then treated with glutamate at different concentrations (0.25, 0.5, 1, 2, 4, 8, 16 and 32 mM) for 24 h. Then the cell viability was evaluated by resazurin assay. The results were presented as a percentage of the normal control group. Values are the mean ± SD (*n* = 3). Significance: * *p* < 0.05, ** *p* < 0.01 *vs.* normal control group without glutamate.

Both undifferentiated and differentiated rat adrenal medullary phenochromocytoma PC12 cells were purchased from the Cell Bank of the Chinese Academy of Sciences (Shanghai, China). Differentiated PC12 cells can form synapses, which are very similar to human neurons. A previously published resazurin assay [21] was used to measure glutamate-induced PC12 cell death. As shown in Figure 2A, glutamate treatment for 24 h could not induce cell death of undifferentiated PC12 cells even when glutamate concentration was as high as 32 mM. However, glutamate induced the death of differentiated PC12 cells in a concentration-dependent manner (Figure 2B), indicating glutamate-induced death was PC12 cell differentiating-dependent.

Glutamate treatment of the differentiated PC12 cells at a concentration of 4 mM led to about 50.0% cell death, thus glutamate at 4 mM was chosen for the subsequent experiments.

### 2.3. Oligosaccharides as Antagonists against Glutamate-Induced PC12 Cell Death

**Figure 3:**
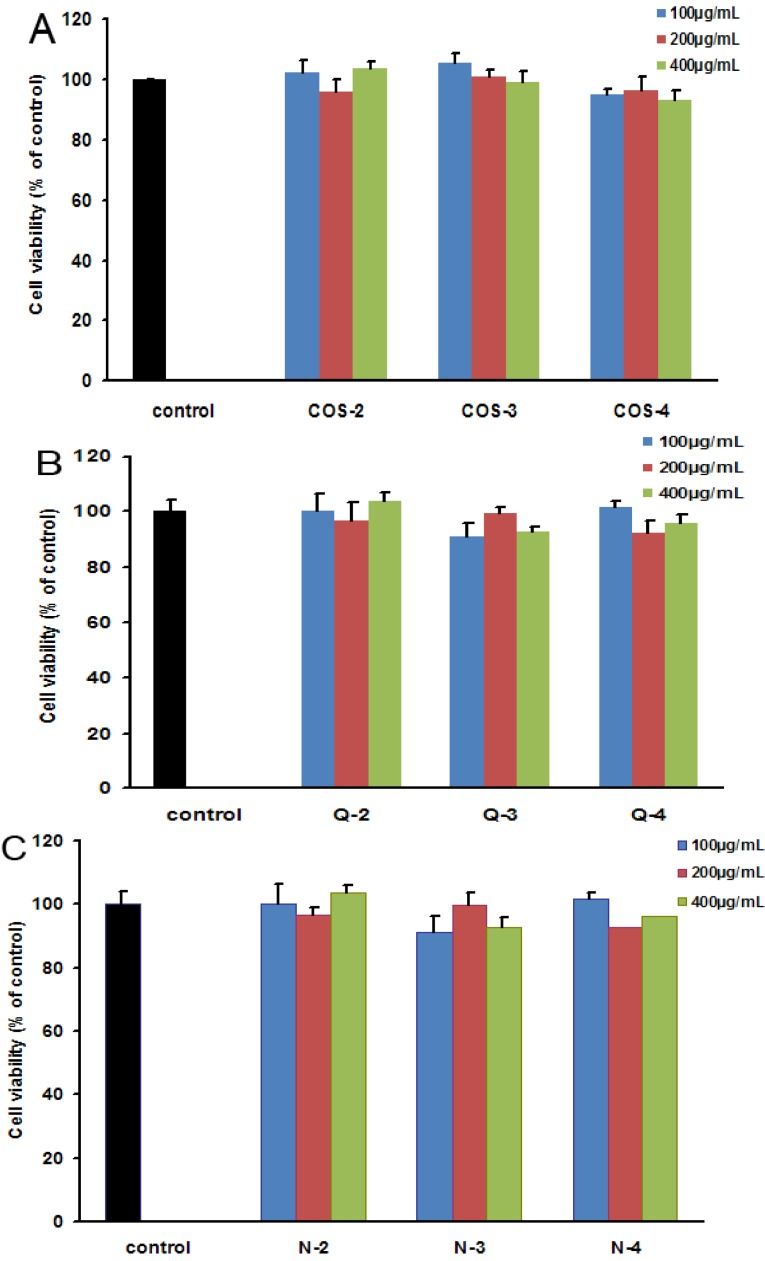
The cytotoxicity of different chitosan oligosaccharides in PC12 cells. PC12 cells were incubated with different chitosan oligosaccharides COS-2~4 (**A**), peracetylated chitosan oligosaccharides Q-2~4 (**B**) and *N*-acetylated chitosan oligosaccharides N-2~4 (**C**) at indicated concentrations (100, 200, 400 μg/mL) for 24 h. Then the cell viability was evaluated by resazurin assay. The results were presented as a percentage of non drug treated normal control group. Values are the mean ± SD (*n* = 3).

Chitosan oligosaccharides and their acetylated derivatives were first tested for their cytotoxicities in fully differentiated PC12 cells by resazurin assay as described previously [21,22,23], and the results indicated that none of chitosan oligosaccharides and their acetylated derivatives (COSs, PACOs and NACOs) exhibited any significant cytotoxicity even at the concentration of 400 μg/mL (Figure 3). The low cytotoxicity suggested that chitosan oligosaccharides and their acetylated derivatives were ideal compounds to use for screening antagonists against glutamate-induced cell death in PC12 cells.

**Figure 4:**
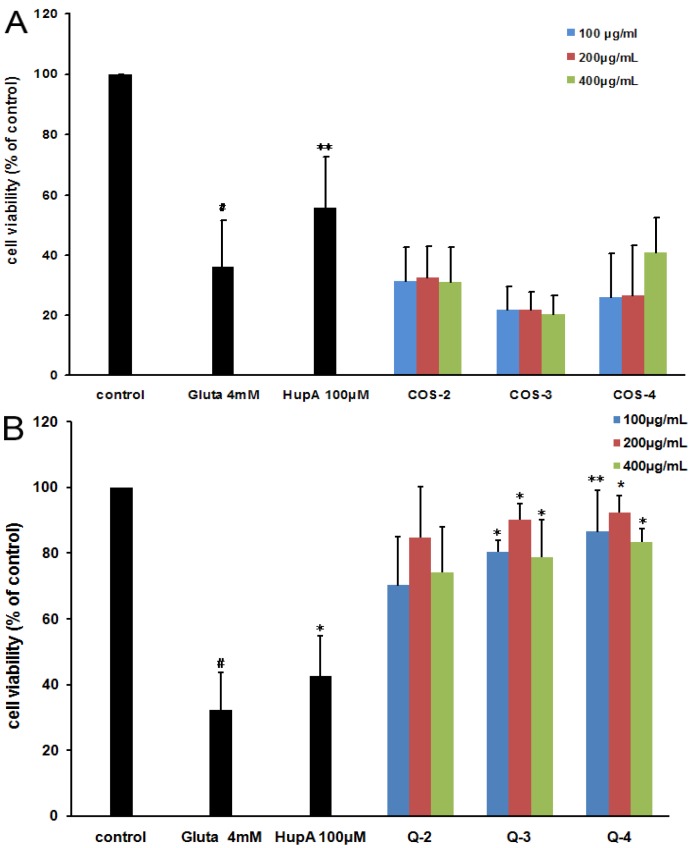
The effect of different chitosan oligosaccharides on glutamate-induced PC12 cell damage. PC12 cells were treated with or without different chitosan oligosaccharides COS-2~4 (**A**) and peracetylated chitosan oligosaccharides Q-2~4 (**B**) at indicated concentrations for 2 h. Then cells were treated with glutamate for another 24 h before performing a resazurin assay. The untreated normal cells (control) were assigned values of 100 and the results presented as mean ± SD (*n* = 4). Significance: # *p* < 0.05 *vs.* normal control group; * *p* < 0.05, ** *p* < 0.01 *vs.* glutamate treated control group.

These compounds as antagonists against glutamate-induced fully differentiated PC12 cell death were then evaluated by resazurin assay. As shown in Figure 4B, the cells that were exposed to 4 mM l-glutamate showed a significant decrease in cell viability (32% ± 11%, relative to the untreated control group). However, pretreatment with peracetylated chitosan oligosaccharides Q3 or Q4 at 100, 200, and 400 μg/mL for 2 h before l-glutamate exposure significantly restored cell viability, which ranged from about 30% to about 85% as compared to control cells (*p* < 0.01), which was superior to the effect of the positive control drug Huperzine-A (HupA, 100 μM) (about 45%) (Figure 4B). The cell death-preventing effect at 200 μg/mL was greater than the other two concentrations tested (100 and 400 μg/mL) (Figure 4B). In contrast, the non-acetylated chitosan oligosaccharides (COS-2, COS-3, and COS-4) had almost no antagonist effects against glutamate-induced PC12 cell death (Figure 4A). These results indicated that the acetylation modification of chitosan oligosaccharides enhanced their antagonist effects against glutamate-induced PC12 cell death.

### 2.4. The Structure-Activity Relationship of Acetylated Chitosan Oligosaccharides

**Figure 5:**
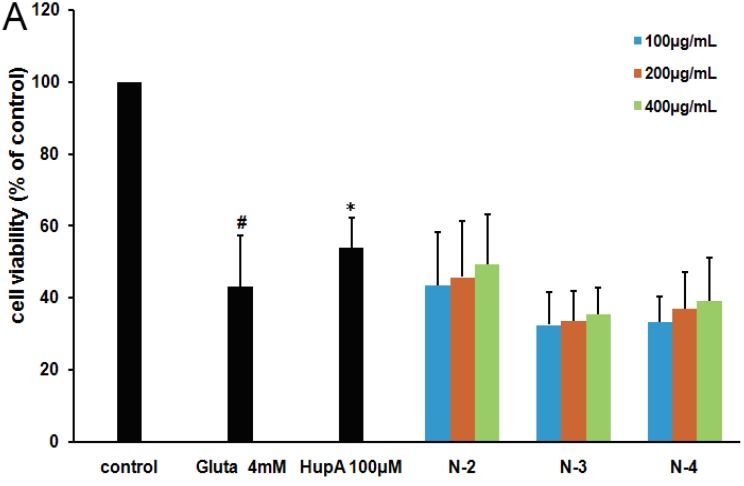

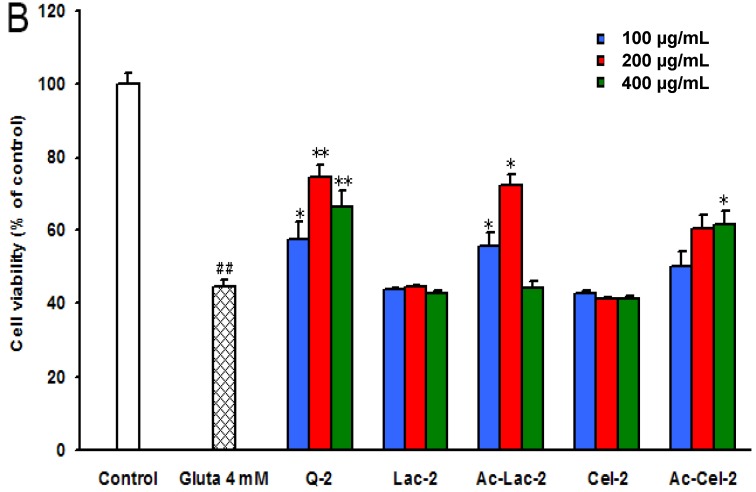
The neuroprotective effects of different acetylated neutral oligosaccharides in PC12 cells. (**A**) PC12 cells were treated with or without different N-acetylated chitosan oligosaccharides N-2~4 at indicated concentrations for 2 h. Then cells were treated with glutamate for another 24 h before performing a resazurin assay. The untreated normal cells (control) were assigned values of 100 and the results presented as mean ± SD (*n* = 4). Significance: # *p* < 0.05 *vs.* normal control group; * *p* < 0.05 *vs.* glutamate treated control group. (**B**) PC12 cells were treated with or without peracetylated chitobiose Q-2, lactose, acetylated lactose, cellobiose, or acetylated cellobiose at indicated concentrations for 2 h. Then cells were treated with glutamate for another 24 h. The untreated normal cells (control) were assigned values of 100 and the results presented as mean ± SD (*n* = 3). Significance: ## *p* < 0.01 *vs.* normal control group; * *p* < 0.05, ** *p* < 0.01 *vs.* glutamate treated control group.

The influence of molecular weight and the location of the acetylation group on chitosan oligosaccharides were investigated by performing a resazurin assay. As shown in Figure 4B, the peracetylated chitotetraose Q-4 had the best neuroprotective effect among Q-2, Q-3, Q-4, which suggested that the higher molecular weight of peracetylated chitosan oligosaccharides correlated with superior antagonist activity. However, there was no significant difference in the neuroprotective effects of those acetylated chitosan oligosaccharides (*n* = 0, 1, 2) (Figure 4B), which suggested that the degree of polymerization (DP) is not the key factor for their neuroprotective effects. Interestingly, the *N*-acetylated chitosan oligosaccharides with a degree of acetylation of 1.0 had no significant antagonist effect (Figure 5A), which suggested peracetylation was essential for the antagonist effect of chitosan oligosaccharides against glutamate-induced PC12 cell death.

Furthermore, to explore whether the acetyl group was indispensible for the neuroprotective effect of the oligosaccharides, we made another two peracetylated oligosaccharides and evaluated their neuroprotective effects in PC12 cells. As shown in Figure 5B, the acetylated chitobiose Q-2 had the best neuroprotective effect among these acetylated oligosaccharides, and the effect at 200 μg/mL was better than that at other two concentrations tested (100 and 400 μg/mL). Interestingly, the non-acetylated oligosaccharides lactose Lac-2 and cellobiose Cel-2 had no significant antagonist effect against glutamate-induced cell death (Figure 5B) resembling non-acetylated chitosan oligosaccharides. However, after acetylation, the neuroprotective effects of peracetylated lactose Ac-Lac-2 and peracetylated cellobiose Cel-2 were all greater than that of non-acetylated oligosaccharides Lac-2 and Cel-2, and the effect of Ac-Lac-2 at 200 μg/mL was comparable to that of Q-2 (Figure 5B). These results suggested that the acetyl group is indispensible for the neuroprotective effect of the oligosaccharides, and the structure of the sugar backbone might also influence the antagonist effect against glutamate-induced PC12 cell death.

### 2.5. Effect of Peracetylated Chitosan Oligosaccharides (PACO) on LDH Release and ROS Production

**Figure 6:**
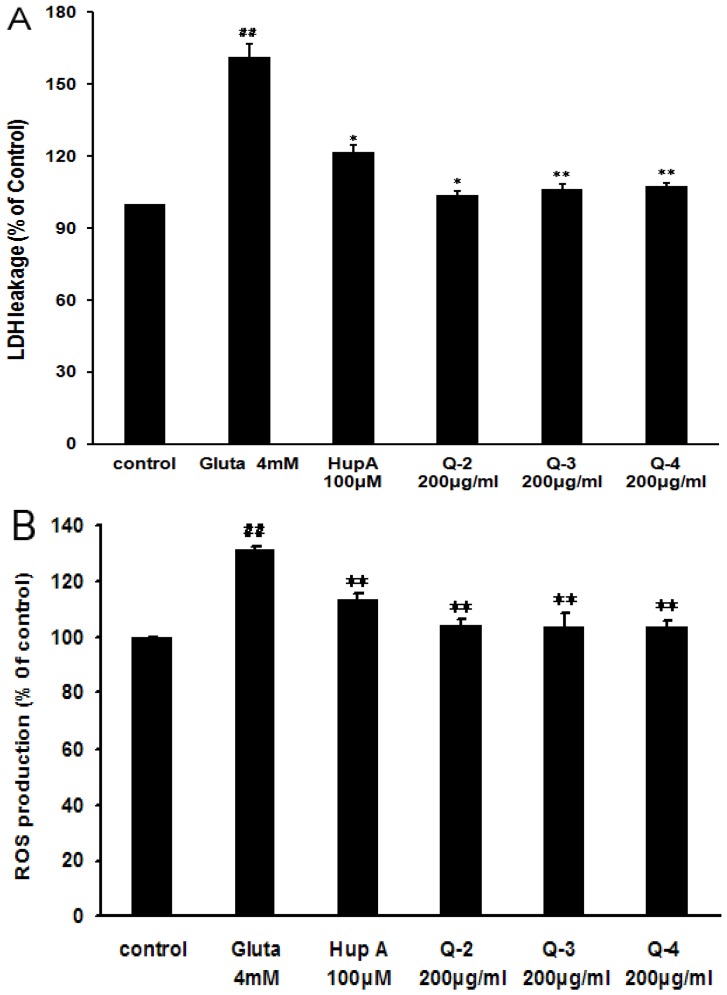
Effect of peracetylated chitosan oligosaccharides on glutamate-induced LDH release and ROS overproduction. (**A**) After the treatment of cells with different PACO monomers Q-2, Q-3 and Q-4 at a concentration of 200 μg/mL for 2 h and 4 mM of glutamate for 24 h, the level of LDH in the culture media was measured using an LDH assay kit. The data were normalized to the activity of LDH released from control cells. Values are the mean ± SD (*n* = 4). Significance: ## *P* < 0.01 *vs.* normal control group; * *P* < 0.05, ** *P* < 0.01 *vs.* glutamate treated control group; (**B**) After the treatment of cells with different PACO monomers Q-2, Q-3 and Q-4 at the concentration of 200 μg/mL for 2 h and 4 mM of glutamate for another 24 h. The fluorescence intensity of DCF was measured in a microplate-reader. Data were expressed as a percentage of non-treated control. Values are the mean ± SD (*n* = 4). Significance: ## *P* < 0.01 *vs.* normal control group; ** *P* < 0.01 *vs.* glutamate treated control group.

To further investigate the underlying molecular mechanisms of acetylated chitosan oligosaccharides as antagonists against glutamate-induced PC12 cell death, the LDH release assay, another indicator of cell toxicity, was performed. As shown in Figure 6A treatment with glutamate (4 mM) resulted in an increase of LDH release into the medium, which was 161% ± 3% as compared to control cells (Figure 6A). Pre-incubation with peracetylated chitosan oligosaccharides Q3, Q4 at the concentration of 200 μg/mL significantly blocked LDH leakage in the PC12 cell system (*p* < 0.01), which was decreased from 161% to about 105% as compared to control cells (Figure 6A). The inhibition effect of peracetylated chitosan oligosaccharides Q2, Q3, and Q4 on LDH release was greater than that of the positive control drug, huperzine A (HupA, 100 μM) (Figure 6A).

Furthermore, the degree of ROS accumulation after glutamate exposure was also measured to determine the role of ROS in glutamate-induced PC12 cell death. As shown in Figure 6B, treatment with glutamate (4 mM) resulted in an increase of ROS in PC12 cells, which was about 131% ± 5% as compared to control cells. However, pre-treatment with peracetylated chitosan oligosaccharides Q2, Q3, and Q4 at the concentration of 200 µg/mL significantly reduced glutamate induced ROS production from 131% to 103% (*p* < 0.01), which was superior to the effect of positive control drug huperzine A (HupA, 114%) (Figure 6B).

### 2.6. Effects of PACO on Mitochondrial Membrane Potential and the Activation of Caspase-3 and Caspase-9

**Figure 7:**
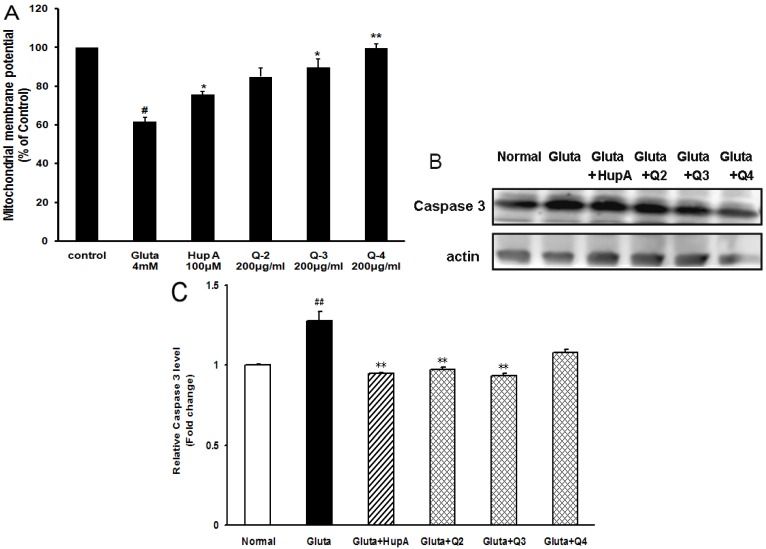

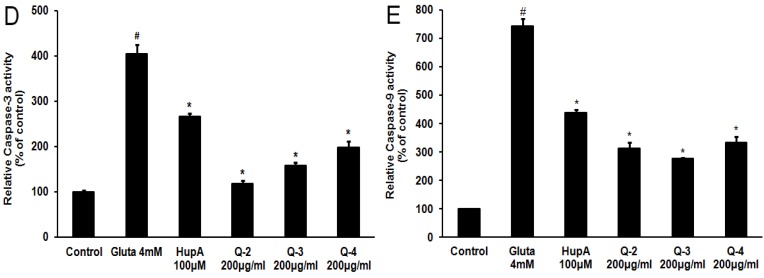
Peracetylated chitosan oligosaccharides protect PC12 cells against glutamate-induced loss of MMP and the activation of Caspase-3 and Caspase-9. (**A**) After being pretreated with 200 μg/mL of different PACO monomers Q-2, Q-3 and Q-4 for 2 h and 4 mM of glutamate for another 24 h, the mitochondrial membrane potential (MMP) in PC12 cells were evaluated with the probe JC-1. Data were expressed as a percentage of non-treated control. Values are the mean ± SD (*n* = 3). Significance: # *p* < 0.05 *vs.* normal control group; * *p* < 0.05, ** *p* < 0.01 *vs.* glutamate treated control group; (**B**) After being pretreated with 200 μg/mL of different PACO monomers Q-2, Q-3 and Q-4 for 2 h and 4 mM of glutamate for another 24 h, the levels of cleaved caspase-3 were measured by western blot. Blots were also probed for β-actin protein as loading controls. The result shown is a representative of three separate experiments with similar results. (**C**) Quantification of immunoblot for the ratio of caspase-3 to β-actin. The ratio for non-treated normal control cells was assigned values of 1.0 and the data presented as mean ± SD (*n* = 3). Significance: ## *p* < 0.01 *vs.* normal control group; ** *p* < 0.01 *vs.* glutamate treated control group. (D–E) After treatment, the activities of caspase-3 (**D**) and caspase-9 (**E**) were measured using an ELISA assay kit (Beyotime, China). Data were expressed as a percentage of non-treated control. Values are the mean ± SD (*n* = 3). Significance: # *p* < 0.05 *vs.* normal control group; * *p* < 0.05 *vs.* glutamate treated control group.

Since glutamate-induced ROS production was a mitochondria-associated event, the changes in mitochondria induced by glutamate in the presence or absence of the PACOs were then tested. We observed the collapse of mitochondrial membrane potential (MMP) in PC12 cells with the probe JC-1. In brief, after incubation of PC12 cells with glutamate (4 mM) for 24 h, JC-1 uptake was measured by MMP assay kit according to the methods described previously [24]. As shown in Figure 7A, the MMP decreased to 62% ± 2% of control after glutamate treatment. Pretreatment with different PACO monomers (Q-3 and Q-4) at a concentration of 200 μg/mL could significantly protected cells against the glutamate-induced lowering of MMP from 62% to about 90% (*p* < 0.05), which was superior to the effect of the positive control drug, HupA (about 75%) (Figure 7A). The PACO monomer Q-2 could also inhibit the loss of MMP although insignificantly (Figure 7A). These results suggested that the PACOs might protect PC12 cells against glutamate induced apoptosis by attenuating the loss of mitochondrial membrane potential.

Caspases are crucial proteases that drive apoptosis. In our study, caspase-3 was significantly activated in PC12 cells, *i.e.*, 1.3-fold higher than normal control group after treatment with 4 mM of glutamate for 24 h (Figure 7B). However, pretreatment with peracetylated chitosan oligosaccharides (Q-2, Q-3 and Q-4) at the concentration of 200 μg/mL prior to exposure to glutamate could decrease the expression level of caspase 3 proteins from 1.3 to about 0.9, 0.9 and 1.0 fold of normal control group, respectively (Figure 7B,C). Moreover, the activities of caspase-3 and caspase-9 were also evaluated by ELISA assay, and the results indicated that pretreatment with PACOs (Q-2, Q-3 and Q-4) could significantly decrease the activities of both caspase-3 and caspase-9 in PC12 cells (*p* < 0.05), which were superior to the effect of positive control drug HupA (Figure 7D,E). These data suggested that the PACO may inhibit glutamate-induced cell death by down-regulating caspase-3 protein.

### 2.7. Effects of PACO on the Cytochrome c (Cyto C) Release from Mitochondria

**Figure 8:**
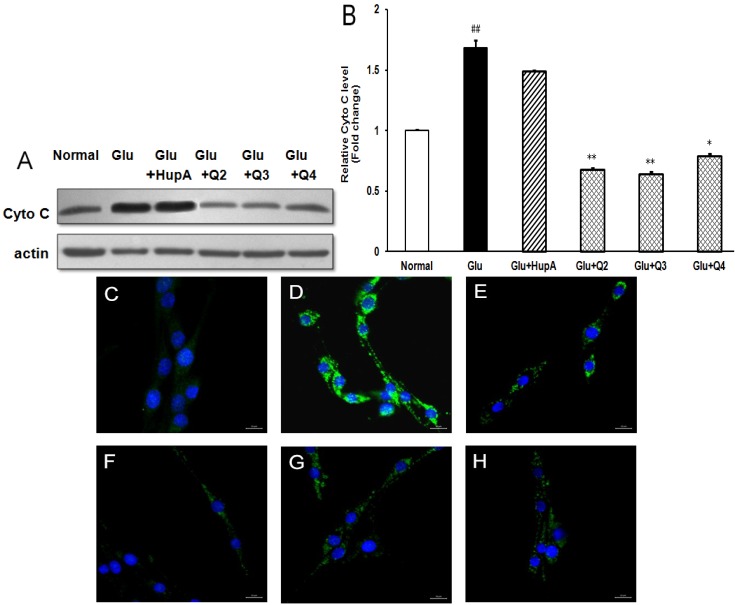
Peracetylated chitosan oligosaccharides protect PC12 cells against glutamate-induced Cyto C release from mitochondria. (**A**) After being pretreated with 200 μg/mL of different PACO monomers Q-2, Q-3 and Q-4 for 2 h and 4 mM of glutamate for another 24 h, the protein levels of Cyto C were evaluated by western blot. Blots were also probed for β-actin as loading controls. The result shown is a representative of three separate experiments with similar results; (**B**) Quantification of immunoblot for the ratio of Cyto C to β-actin. The ratio for non-treated control cells was assigned values of 1.0 and the data presented as mean ± SD (*n* = 3). Significance: ## *p* < 0.01 *vs.* normal control group; * *p* < 0.05, ** *p* < 0.01 *vs.* glutamate treated control group. (**C**–**H**) After being pretreated with 200 μg/mL of PACO monomers Q-2, Q-3 and Q-4 for 2 h, PC12 cells were exposed to 4 mM glutamate for 24 h. Then the levels of Cyto C in the cytoplasm were detected by immunofluorescence assay using anti-Cyto C antibody. **C**: Normal control, **D**: Glu, **E**: Glu + HupA, **F**: Glu + Q2, G: Glu + Q3, **H**: Glu + Q4. Scale bar represents 20 μm.

Previous study has shown that cytochrome c (Cyto C) release from mitochondria triggers the apoptotic program [25]. Therefore, we further investigated the possible effect of the PACOs on glutamate-induced cytochrome c release from mitochondria by western blotting assay. As shown in Figure 8A,B, 4 mM glutamate caused significant cytochrome c release, which was 1.7-fold higher than normal control group. However, pretreatment with peracetylated chitosan oligosaccharides (Q-2, Q-3 and Q-4) at a concentration of 200 μg/mL prior to exposure to glutamate could significantly decrease the protein level of Cyto C proteins from 1.7 to about 0.7, 0.6 and 0.8 fold of normal control group, respectively (*p* < 0.05), which was superior to the effect of positive control drug HupA (about 1.4 fold) (Figure 8A,B). These data suggested that the PACO may inhibit cytochrome c release from mitochondria.

To further explore the effects of PACO on Cyto C release from mitochondria, the protein level of cytochrome c in cytoplasm was also measured by immunofluorescence assay. As shown in Figure 8C–H, treatment with glutamate (4 mM) resulted in an increase of cytochrome c in cytoplasm (Figure 8D), while there were nearly no cytochrome c release in normal control cells (Figure 8C). However, pre-treatment with peracetylated chitosan oligosaccharides Q2, Q3, and Q4 at the concentration of 200 µg/mL significantly reduced the protein level of cytochrome c in cytoplasm (Figure 8F–H), which was better than the effect of positive control drug huperzine A (Figure 8E). In summary, PACOs may inhibit glutamate-induced cell death by inhibiting cytochrome c release from mitochondria.

### 2.8. Effects of PACO on the Protein Expression of Bcl-2 and Bax

**Figure 9:**
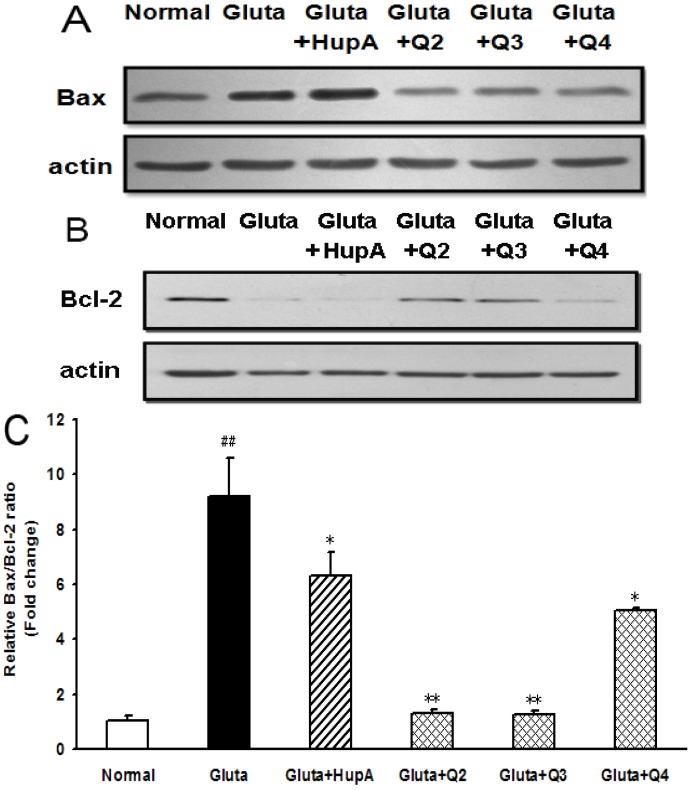
Effect of PACOs on the expression of Bax and Bcl-2 in PC12 cells. (A–B) After being pretreated with 200 μg/mL of different PACO monomers Q-2, Q-3 and Q-4 for 2 h and 4 mM of glutamate for another 24 h, the levels of Bax (**A**) and Bcl-2 (**B**) were measured by western blot. Blots were also probed for β-actin as loading controls. The result shown is a representative of three separate experiments with similar results; (**C**) Quantification of immunoblot for the ratio of Bax and Bcl-2. The ratio for non-treated normal control cells was assigned values of 1.0. Values are the mean ± SD (*n* = 3). Significance: ## *p* < 0.01 *vs.* normal control group; * *p* < 0.05, ** *p* < 0.01 *vs.* glutamate treated control group.

To determine whether Bcl-2 and Bax participated in the process of glutamate-induced PC12 cell death, the cells were treated with or without different drugs and then the expression levels were examined by western blot analysis. As shown in Figure 9A, Bax expression was markedly increased in the glutamate-treated group as compared to normal control group. However, pretreatment with 200 µg/mL of peracetylated chitosan oligosaccharides (Q-2, Q-3 and Q-4) prior to exposure to glutamate significantly decreased the expression level of Bax protein compared to the glutamate-treated control group (Figure 9A). In contrast, the level of Bcl-2 in the glutamate-treated group decreased significantly compared to normal control group (Figure 8B). However, Bcl-2 expression was ameliorated after the PACO treatment in PC12 cells and the effects of Q-2 and Q-3 were better than that of Q-4 and HupA (Figure 9B).

Moreover, the Bax/Bcl-2 ratio after glutamate-treatment increased to levels that were about 9.2-fold higher than normal control group (Figure 9C), suggesting glutamate induced apoptosis of PC 12 cells. Treatment with peracetylated chitosan oligosaccharides (Q-2, Q-3 and Q-4) decreased the Bax/Bcl-2 ratio significantly at a concentration of 200 μg/mL (*p* < 0.05), and they altered the Bax/Bcl-2 ratio from about 9.2 to about 1.3, 1.2 and 5.0, respectively, when compared to that of normal control group (Figure 9C). The inhibition effect of peracetylated chitosan oligosaccharides Q2, Q3, and Q4 on Bax/Bcl-2 ratio was better than that of positive control drug HupA (about 6.0 fold) (Figure 9C). Taken together, these results suggested that the protective effect of the PACO on glutamate-induced apoptosis in PC12 cells might be through Bcl-2/Bax signal pathway.

**Figure 10:**
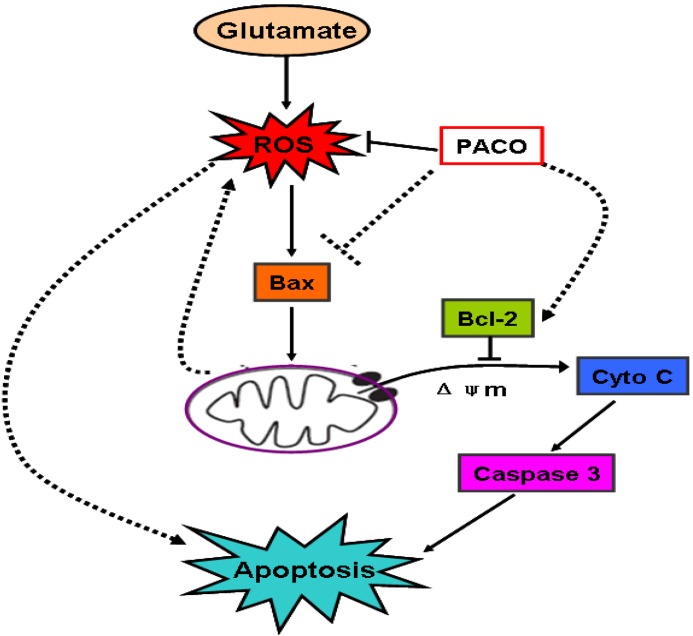
Proposed schemas of the mechanisms by which PACO suppressed glutamate-induced apoptosis in PC12 cells. Glutamate induces intracellular ROS generation in PC12 cells. The resultant oxidative stress triggers the activation of Bax protein, thereby reducing ΔΨm, releasing cytochrome c, activating caspase 3, and finally inducing apoptosis. PACO inhibits ROS and suppresses the activity of downstream molecules, such as Bax, Cyto c, and caspase 3, for promoting neuronal survival.

## 3. Experimental Section

### 3.1. Reagents

Chitosan with degree of deacetylation of >95% was purchased from Jinhu Crust Product Corp. (Zibo, China). The lactate dehydrogenase (LDH) assay kit, reactive oxygen species (ROS) detection kit, and mitochondrial membrane potential (MMP) detection kit were purchased from the Jiancheng Institute of Biological Engineering (Nanjing, China). Antibodies for detecting Bcl-2, Bax, Cyto C, and cleaved caspase-3 were obtained from Cell Signaling Technology, Inc. (Danvers, MA, USA). FITC labeled secondary antibodies were purchased from Boster (Wuhan, China). All other reagents were from Sigma-Aldrich (St. Louis, MO, USA) unless otherwise stated.

### 3.2. COSs Production and Purification

The COS mixture was prepared by enzymatic hydrolysis of chitosan according to our previously reported method [19]. In brief, chitosan (50 g) was added to 400 mL distilled water, then 7 mL chitosanase solution (10 U/mL) was added. The mixture was stirred for 24 h in a 50 °C bath, and pH of the reaction mixture was adjusted to 6 by HCl solution (4 mol/L) during the hydrolysis process. The hydrolyzate was adjusted to pH 8~9 with concentrated NaOH and subsequently filtered to remove insoluble parts and the filtrate was concentrated and precipitated by adding four-fold volume of ethanol at 4 °C overnight. The precipitate was collected by centrifugation for 15 min at 8000 rpm then lyophilized to yield powdered products and identified as the COS mixture.

The COS mixture (300 mg) was dissolved in 2 mL of 0.1 M NH_4_HCO_3_, and then filtered with a microporous membrane (0.45 µm) to obtain a clear solution. The filtrate was loaded on a Bio Gel P6 column (2.6 × 110 cm) that connected to an AKTA UPC100 purification system (Fairfield, GE, USA) equipped with an online refractive index detector. The column was eluted with 0.1 M NH_4_HCO_3_ solution at a flow rate 0.4 mL/min. Eluents (10 mL/tube) were collected using a fraction collector to afford the pure dimer, trimer and tetramer of COSs. The analysis of each COS was performed by TLC, HPLC, IR, NMR and MS, as reported in previous work [19]. 

### 3.3. PACOs and NACOs Preparation and Purification

The acetylation of the COSs mixture to prepare PACOs and NACOs was performed according to the reported methods [20] with some modification. 

Dried COSs mixture (5 g) was suspended in acetic anhydride (80 mL) at room temperature with stirring, and 8 mL concentrated H_2_SO_4_ was added. After stirring for 8 h at 50 °C, a clear dark amber-coloured solution was obtained and TLC (CH_2_Cl_2_:CH_3_OH, 12:1, *v*/*v*) indicated the completion of the acetylation. The reaction mixture was added into 100 mL 15% sodium acetate solution at 0 °C and extracted with CH_2_Cl_2_ (80 mL × 3). The organic layer was then dried over anhydrous sodium sulphate, and concentrated under reduced pressure to give a residue of PACOs mixture. The residue was applied to a silica gel column chromatography (CH_2_Cl_2_–CH_3_OH, 50:1 ~ 30:1) to give pure dimer, trimer and tetramer of PACOs. The yield of acetylation of COSs mixture was more than 60%.

The pure dimer, trimer or tetramer of PACOs (2 g) was added to methanol (20 mL). Deacetylation was started by adding sodium methoxide until pH 9~10. The resulting reaction mixture was stirred for 20 h at room temperature and monitored by TLC (propanol: water, 2:1, *v*/*v*). The reaction was then neutralized by addition of ion-exchange resin (H^+^). After filtration, the filtrate was concentrated in vacuum to give pure dimer, trimer or tetramer of NACOs quantitatively.

### 3.4. MS and NMR Spectroscopy of Isolated PACOs and NACOs

Mass spectra were recorded with Micromass Global Q-TOF Mass Spectrometer (Indian Trail, NC, USA) using the ESI technique to determinate the molecular mass of each oligosaccharide. ^1^H and ^13^C NMR spectra were recorded at 27 °C on a JEOL JNM-ECP 600 MHz and an Agilent 500 MHz DD2 spectrometers with tetramethylsilane (Me_4_Si) as the internal standard and chemical shifts were recorded as δ values.

Dimer of PACOs (**Q-2**): ^1^H-NMR (600 MHz, CDCl_3_): δ 6.17 (d, 1H, *J* = 9.2 Hz, N-H′), 6.10 (d, 1H, *J* = 3.6 Hz, H-1), 5.82 (d, 1H, *J* = 9.0 Hz, N-H), 5.23 (dd, 1H, *J* = 11.1, 9.0 Hz, H-3), 5.14 (dd, 1H, *J* = 9.4, 10.4 Hz, H-3′), 5.07 (t, 1H, *J* = 9.6 Hz, H-4′), 4.50 (d, 1H, *J* = 8.4 Hz, H-1′), 4.44 (dd, 1H, *J* = 12.3, 3.8 Hz, H-6-1), 4.40 (dd, 1H, *J* = 12.6, 4.1 Hz, H-6-1′), 4.40–4.35 (m, 1H, H-2), 4.21 (dd, 1H, *J* = 2.1, 12.1Hz, H-6-2), 4.02 (dd, 1H, *J* = 2.1, 12.4Hz, H-6-2′), 3.97 (app q, 1H, *J* = 9.2 Hz, H-2′), 3.91 (ddd, 1H, *J* = 2.2, 3.4, 10.1 Hz, H-5), 3.76 (t, 1H, *J* = 9.5Hz, H-4), 3.65 (ddd, 1H, *J* = 2.4, 4.0, 9.9Hz, H-5′), 2.19, 2.15, 2.09, 2.06, 2.01, 2.01,(6s, 18H, 3×-OCOCH_3_), 1.96, 1.94 (2s, 6H, 2×-NHCOCH_3_). ^13^C NMR (150 MHz, CDCl_3_): δ 171.4, 171.2, 170.7, 170.5 170.4, 170.2, 169.2, 168.9 (8C=O), 101.7 (C-1′), 90.4 (C-1), 75.9 (C-4), 72.5(C-3′), 71.8(C-5′), 70.7 (C-3), 70.6(C-5), 67.8 (C-4′), 61.6 (C-6′), 61.4 (C-6), 54.3 (C-2′), 51.0 (C-2), 23.1, 23.0 (2CH_3_CONH), 21.0, 20.9, 20.6, 20.6, 20.5, 20.5 (6CH_3_COO). ESI-MS *m*/*z* 699.1 [M + Na]^+^. 

Trimer of PACOs (**Q-3**): ^1^H-NMR (500 MHz, DMSO-d6): δ 7.97 (d, 1H, *J* = 9.1 Hz, N-H), 7.91 (d, 1H, *J* = 9.2 Hz, N-H), 7.91 (d, 1H, *J* = 9.4 Hz, N-H), 5.81 (d, 1H, *J* = 3.5 Hz, H-1), 5.12 (t, 1H, *J* = 9.9 Hz), 5.05–4.97 (m, 2H), 4.81 (t, 1H, *J* = 9.7 Hz), 4.64 (d, 1H, *J* = 8.4 Hz, H-1″), 4.55 ( d, 1H, *J* = 8.4 Hz, H-1′), 4.36–4.25 (m, 3H), 4.15–4.10 (m, 1H), 4.05–4.00 (m, 2H, H-2), 3.91–3.77 (m, 4H), 3.72 (t, 1H, *J* = 9.3 Hz), 3.60–3.48 (m, 3H), 2.15, 2.09, 2.05, 2.00, 1.95, 1.94(2CH_3_), 1.90 (7s, 24H, 8 × OCOCH_3_), 1.78, 1.74, 1.73 (3s, 9H, 3 × NHCOCH_3_); ^13^C NMR (125 MHz, DMSO-d6): δ 170.1, 170.0, 169.9, 169.8, 169.6, 169.5, 169.4, 169.3, 169.2, 169.1 (11C=O), 100.1, 99.9 (C-1′, C-1″), 89.6 (C-1), 75.6, 74.8, 73.3, 72.3, 71.6, 70.4, 70.1(2C), 68.1, 62.6, 61.6, 61.5, 53.8, 53.7, 50.0, 22.6, 22.5, 22.2(3CH_3_CONH), 20.8–20.3 (m, 8CH_3_COO). ESI-MS *m*/*z* 986.2 [M + Na]^+^.

Tetramer of PACOs (**Q-4**): ^1^H-NMR (500 MHz, DMSO-d6): δ 7.96 (d, 1H, *J* = 9.1 Hz, N-H), 7.92–7.87 (m, 3H, 3NH), 5.81 (d, 1H, *J* = 3.5 Hz, H-1), 5.12 (t, 1H, *J* = 9.9 Hz), 5.05–4.94 (m, 3H), 4.81 (t, 1H, *J* = 9.7 Hz), 4.63 (d, 1H, *J* = 8.4 Hz, H-1‴), 4.55 (d, 2H, *J* = 8.4 Hz, H-1″, H-1′), 4.36–4.24 (m, 4H), 4.14–3.99 (m, 4H), 3.90–3.65 (m, 6H), 3.60–3.45 (m, 5H), 2.14, 2.08, 2.07, 2.04, 2.00, 1.95, 1.93(2CH_3_), 1.89 (8s, 30H, 10 × OCOCH_3_), 1.78, 1.74, 1.72 (3s, 12H, 4 × NHCOCH_3_); ^13^C NMR (125 MHz, DMSO-d6): δ 170.1, 170.1, 170.0, 169.9, 169.8, 169.6, 169.5, 169.4, 169.3, 169.3, 169.2, 169.1 (14C=O), 100.1, 99.9, 99.8 (C-1′, C-1″, C-1‴), 89.6 (C-1), 75.5, 75.2, 74.7, 73.2, 73.1, 72.3, 71.7, 71.5, 70.4, 70.2, 70.1, 68.1, 62.5 (2C), 61.6, 61.5, 53.9, 53.8, 53.7, 50.0, 22.6, 22.6, 22.5, 22.2(4CH_3_CONH), 20.8–20.3 (m, 10CH_3_COO). ESI-MS *m*/*z* 1251.6 [M + H]^+^.

Dimer of NACOs (**N-2**): ^1^H-NMR (600 MHz, D_2_O): δ 5.16 (d, 0.6H, *J* = 2.8 Hz, H-1α), 4.66 (d, 0.4H, *J* = 7.9 Hz, H-1β), 4.56 (d, 0.6 H, *J* = 8.5 Hz, H-1′α), 4.55 (d, 0.4 H, *J* = 8.4 Hz, H-1′β), 3.90–3.43 (m, 12H, H-2, H-3, H-4, H-5, H-6-1, H-6-2, H-2′, H-3′, H-4′, H-5′, H-6-1′, H-6-2′), 2.04, 2.00 (2s, 6H, 2 × -NHCOCH_3_); ^13^C NMR (150 MHz, D_2_O): δ 174.9, 174.7 (2C=O), 101.8 (C-1′), 95.1 (C-1β), 90.7 (C-1α), 80.1(C-4α), 79.6 (C-4β), 76.2 (C-5′), 74.8 (C-5β), 73.7 (C-3′), 72.8 (C-3β), 70.2 (C-5α), 70.0 (C-4′), 69.5 (C-3α), 60.8 (C-6′), 60.4 (C-6β), 60.3 (C-6α) 56.3 (C-2β), 55.9 (C-2′), 53.9 (C-2α), 22.4, 22.2 (2CH_3_). ESI-MS *m*/*z* 425.2 [M + H]^+^.

Trimer of NACOs (**N-3**): ^1^H-NMR (600 MHz, D_2_O): δ 5.15 (d, 0.65 H, *J* = 2.6 Hz, H-1α), 4.66 (d, 0.36 H, *J* = 7.9 Hz, H-1β), 4.56 (d, 0.65 H, *J* = 8.2 Hz, H-1′), 4.55 (d, 0.65 H, *J* = 8.5 Hz, H-1″), 3.90–3.43 (m, 18H, H-2~H-6, H-2′~H-6′, H-2″~H-6″), 2.03, 2.03 2.01( 3s, 9H, 3 × -NHCOCH_3_). ^13^C NMR (150 MHz, D_2_O): δ 174.9, 174.9, 174.7 (3C=O), 101.8(C-1″), 101.6 (C-1′), 95.1 (C-1β), 90.7 (C-1α), 79.9 (C-4α), 79.4 (C-4′, C-4β), 76.2 (C-5″), 74.9 (C-5β), 74.8 (C-5′), 73.7 (C-3″), 72.8 (C-3β), 72.4 (C-3′), 70.3 (C-5α), 70.0 (C-4″), 69.5 (C-3α), 60.8 (C-6″), 60.4 (C-6β), 60.3 (C-6′, C-6α), 56.4 (C-2β), 55.9 (C-2″), 55.3 (C-2′), 53.9 (C-2α), 22.4, 22.4, 22.2 (3CH_3_). ESI-MS *m*/*z* 628.1 [M + H]^+^.

Tetramer of NACOs (**N-4**): ^1^H-NMR (600 MHz, D_2_O): δ 5.18 (d, 0.64 H, *J* = 1.7 Hz, H-1α), 4.68 (d, 0.35 H, *J* = 8.1 Hz, H-1β), 4.57 (d, 3 H, *J* = 8.4 Hz, H-1′, H-1″, H-1‴), 3.92–3.44 (m, 24H, H-2~H-6, H-2′~H-6′, H-2″~H-6″, H-2‴~H-6″′), 2.05, 1.05, 2.05, 2.03 (4s, 12H, 4 × -NHCOCH_3_). ^13^C NMR (150 MHz, D_2_O): δ 175.0, 174.9, 174.7 (4C=O), 101.7 (C-1‴), 101.5 (C-1′, C-1″), 95.1 (C-1β), 90.7 (C-1α), 79.9 (C-4α), 79.4 (C-4′, C-4″), 79.2 (C-4β), 76.2 (C-5‴), 74.9 (C-5β), 74.8 (C-5′, C-5″), 73.7 (C-3‴), 72.8 (C-3β), 72.4 (C-3′, C-3″), 70.3 (C-5α), 70.0 (C-4‴), 69.5 (C-3α), 60.8 (C-6‴), 60.4 (C-6β), 60.3 (C-6′, C-6″, C-6α), 56.4 (C-2β), 55.9 (C-2‴), 55.3 (C-2′, C-2″), 53.9 (C-2α), 22.5, 22.4, 22.2 (4CH_3_). ESI-MS *m*/*z* 831.2 [M + H]^+^.

### 3.5. Cell Culture

Both undifferentiated and differentiated rat adrenal medullary phenochromocytoma PC12 cells were purchased from the Cell Bank of the Chinese Academy of Sciences (Shanghai, China). Cells were maintained in Dulbecco’s Modified Eagle’s medium (DMEM, Gibco, New York, NY, USA) supplemented with 10% FBS (Gibco, New York, NY, USA), 100 U/mL penicillin and 100 μg/mL streptomycin at 37 °C in 5% CO2 atmosphere. Cells were passaged with trypsin every 4 days with the subcultivation ratio of 1:6. All experiments were performed on cells between passages 3–15.

### 3.6. Determination of Cell Viability

The cell viability of PC12 cells was measured by resazurin assay [21]. In brief, after oligosaccharide treatment for 24 h, 20 μL of resazurin was added to each well at a final concentration of 0.2 mg/mL and incubated for 16 h at 37 °C. Then resazurin fluorescence was measured using a SpectraMax M5 plate reader (Molecular Devices, Sunnyvale, CA, USA) with excitation and emission wavelengths of 544 and 595 nm, respectively. The cell viability was expressed as a percentage of non-treated control. 

For the neuroprotective experiments, PC12 cells were seeded into 96-well plates at a density of 5 × 103 cells/well, and cultivated for 24 h. Cells were pretreated with different oligosaccharides at concentrations of 100, 200, 400 μg/mL or 100 µM of Huperzine-A (HupA) for 2 h prior to exposure to 4 mM of glutamate. After 24 h incubation, cell viability was evaluated by resazurin assay.

### 3.7. LDH Release Assay

The level of lactate dehydrogenase (LDH) released from damaged cells into culture media was measured using an LDH assay kit (Beyotime, Nantong, China) according to the manufacturer’s protocol. Briefly, PC12 cells were seeded at a density of 5 × 103 cells/well into 96-well plate. After drug treatment, the cell-free culture supernatants were collected from each well and incubated with the appropriate reagent mixture according to the supplier’s instructions at room temperature for 20 min. The absorbance of samples was measured at 440 nm using a micro-plate reader. The data were normalized to the activity of LDH released from control cells. Absorbance of blanks, determined as no-enzyme control, had been subtracted from each value.

### 3.8. Measurement of Intracellular ROS

Intracellular ROS was monitored using the DCFH-DA fluorescent probe as described previously [26]. In brief, intracellular H_2_O_2_ or low-molecular-weight peroxides can oxidize DCFH-DA to the highly fluorescent compound dichlorofluorescein (DCF). After drugs treatment, cells were incubated with 10 mM of DCFH-DA at 37 °C for 30 min, and then washed twice with PBS. Finally, the fluorescence intensity of DCF was measured in a micro-plate reader with an excitation wavelength of 485 nm and an emission wavelength of 535 nm. Data were expressed as percentage of non-treated control.

### 3.9. Measurement of the Mitochondrial Membrane Potential (MMP)

The mitochondrial membrane potential (MMP) of PC12 cells was monitored using the fluorescent, lipophilic and cationic probe, JC-1 (Beyotime, Nantong, China) according to the methods described previously [24]. Briefly, after indicated treatments, cells were cultured in 24-well plates and incubated with JC-1 staining solution (5 μg/mL) for 20 min at 37 °C. Cells were then rinsed twice with JC-1 staining buffer and the fluorescence intensity of both mitochondrial JC-1 monomers (λex 514 nm, λem 529 nm) and aggregates (λex 585 nm, λem 590 nm) were detected using a SpectraMax M5 plate reader (Molecular Devices, USA). The Δψm of PC12 cells in each treatment group was calculated as the fluorescence ratio of red (*i.e.*, aggregates) to green (*i.e.*, monomers).

### 3.10. Immunofluorescence Assay

The Cyto C release from mitochondria was also evaluated by using indirect immunofluorescence assay. Briefly, after treatment, cells were fixed with 4% Paraformaldehyde for 15 min at room temperature (RT) and further permeabilized by 0.5% Triton X-100 in PBS for 5 min. Then, after being blocked with 2% bovine serum albumin (BSA) in PBS for 1 h at RT, cells were incubated with anti-Cyto C antibody for 1 h. After three washes with PBS, cells were incubated with FITC labeled secondary antibody for 1 h at RT. Then after washing trice, cells were incubated further with DAPI for 10 min. Finally, the fluorescence corresponding to Cyto C and DAPI was observed by using a laser scanning confocal microscopy (Zeiss LSM510, Oberkochen, Germany).

### 3.11. Western Blot Analysis

Western blot analysis was performed as described previously [27]. Proteins were separated using SDS-PAGE and electrically transferred to a NC membrane (Pall, New York, NY, USA). After that, the membranes were blocked with TBST (50 mM Tris-HCl, pH 7.4, 0.15 M NaCl, 0.1% Tween-20) containing 5% BSA (Sigma, St. Louis, MO, USA) for 2 h. Then the membranes were incubated with primary antibodies against Bax, Bcl-2, or caspase-3 protein diluted at 1:1000 at 4 °C over night. After washing with TBST for three times, the membranes were incubated with goat anti-rabbit IgG labeled with horse radish peroxidase (Santa Cruz, Dallas, TX, USA) diluted at 1:2000 at room temperature for 2 h. Blots were developed using an ECL plus kit (Amersham Bioscience, Aylesbury, UK), exposed to Kodak autoradiographic films and quantified using Image J software. 

### 3.12. Statistical Analysis

All data are represented as the mean ± SD. Comparison between groups was made by one-way analysis of variance (ANOVA) followed by a specific post hoc test to analyze the difference. *p* < 0.05 was considered to indicate statistical significance. The SPSS software package (SPSS program, version 13.0, Chicago, IL, USA) was used for all statistical tests.

## 4. Conclusions

Chitosan is the universally accepted non-toxic *N*-deacetylated derivative of chitin, one of the most abundant biopolymers on earth. Chitosan oligosaccharides (COSs) have been reported to have a variety of biological activities and widely used in many research fields. The current study demonstrated that the peracetylated chitosan oligosaccharides (PACOs) acted as antagonists against glutamate-induced PC12 cell death in a concentration-dependent manner. PACOs significantly prevented glutamate-induced apoptosis as manifested by depressing the elevation of Bax/Bcl-2 ratio and caspase-3 activation, suggesting its antagonist effect could be partially due to apoptosis regulation. Thus, peracetylated chitosan oligosaccharides might have potential for treating certain neurodegenerative diseases.

The antagonist effects of PACOs were greater than that of non-acetylated COSs and *N*-acetylated oligosaccharides NACOs, suggesting that peracetylation was essential for the neuroprotective effects of chitosan oligosaccharides. In addition, the peracetylated lactose Ac-Lac-2 and peracetylated cellobiose Cel-2 both had greater effects than those of the non-acetylated oligosaccharides Lac-2 and Cel-2, but lesser than those of the acetylated chitosan oligosaccharide Q-2, which suggests that the acetyl groups were indispensible for the neuroprotective effect of neutral oligosaccharides and that the structure of the sugar backbone might also influence the neuroprotective effects observed *in vitro*. It was reported that peracetylation can facilitate passive diffusion of disaccharides across cell membranes, allowing them to enter the Golgi [28]. Thus, the acetylation modification might facilitate the chitosan oligosaccharides to enter the cells to interfere the ROS induced apoptosis in PC12 cells.

Glutamate toxicity is an important mechanism of neuronal death in cerebral ischemia [29,30]. It was reported that glutamate neurotoxicity is considered to have two mechanisms of neuron injury including glutamate receptor-mediated [31] and oxidative stress-mediated [32,33] neurotoxicity. In our study, the PACO treatment potentially reduced LDH release and ROS production, and attenuated the loss of mitochondrial membrane potential (MMP), which suggests that the PACOs displayed their protective effect against glutamate-induced apoptosis in PC12 cells mainly through oxidative stress amelioration [34,35,36] (Figure 10).

ROS is a well known etiological factor associated with oxidative stress leading to cell death via apoptosis in a variety of cell types [37,38,39,40], and such effects can be blocked or delayed by a wide variety of antioxidants [41]. Mitochondria-dependent apoptotic pathways are involved in glutamate-induced cytotoxicity in PC 12 cells [42]. Considering the results indicating that PACOs could reduce ROS production and attenuate the loss of MMP, we suppose that the PACOs may be able to protect PC12 cells against glutamate induced cell apoptosis. As a mitochondrial membrane-associated protein, Bcl-2 exerts its anti-apoptotic effect through inhibition of Bax expression from mitochondria, and inhibition of subsequent activation of the caspase-3 [43]. Moreover, cytochrome c release could be reduced by pretreatment with the PACOs, which suggested the PACOs might influence the Bcl-2/Bax pathway because Bax is believed to be upstream of cytochrome c release in the mitochondria-mediated apoptosis pathway. Taken together, glutamate either significantly up- or down-regulated the expression of Bax, Bcl-2 proteins in PC12 cells. Reversal of these trends by the PACO pretreatment suggests that the anti-apoptosis activity of the PACOs may be mediated through Bcl-2/Bax signal pathway in PC12 cells [15,44,45] (Figure 10).

In conclusion, our study demonstrated that the peracetylated chitosan oligosaccharides PACOs exhibited a protective effect on glutamate-induced PC12 cell death through Bcl-2/Bax signal pathway. Since glutamate-evoked cell injury in neuronal cells is involved in many neuron disorders, it thus raises the possibility of developing PACOs as potential agents for prevention and treatment of neurodegenerative diseases. However, PC12 cells cannot totally represent the characteristics of primary cultured neurons, so further work needs to be done directly on primary neurons *in vitro* and on animal models to test if PACOs are useful in treating glutamate-induced neural injury or death.
